# Supplementary material for: Host microbiome depletion attenuates biofluid metabolite responses following radiation exposure
Source: PLoS One. 2024 May 17;19(5):e0300883. doi: 10.1371/journal.pone.0300883 (PMC11101107; doi:10.1371/journal.pone.0300883)
Supplement: S1 Table — (DOCX) [file pone.0300883.s001.docx]

**Supplementary Table 1.** Welch’s t test P-value for urine metabolites that were significantly perturbed due to depletion of host microbiome.

| **Metabolite** | **Welch’s t test**  **P-value** | |
| --- | --- | --- |
| **Urine** | Female | Male |
| Indoxyl Sulfate | <0.001 | 0.008 |
| Pipecolic acid | 0.006 | 0.068 |
| 307.2025_5.69 | <0.001 | <0.001 |
| Hex-V-I | <0.001 | 0.393 |
| N1-Acetylspermidine | 0.001 | 0.003 |
| Niacinamide | <0.001 | 0.111 |
| 3-Methylhistidine | 0.009 | 0.001 |
| Indole-3-acetic acid | <0.001 | 0.001 |
| Hippuric acid | <0.001 | <0.001 |
| TML | <0.001 | 0.501 |
