# Supplementary material for: Host microbiome depletion attenuates biofluid metabolite responses following radiation exposure
Source: PLoS One. 2024 May 17;19(5):e0300883. doi: 10.1371/journal.pone.0300883 (PMC11101107; doi:10.1371/journal.pone.0300883)
Supplement: S2 Table — (DOCX) [file pone.0300883.s002.docx]

**Supplementary Table 2.** Brown-Forsythe P-values for urinary metabolites that were significantly perturbed due to ionizing radiation exposure and significant days identified by Dunnett’s multiple comparisons.

| **Metabolite** | **Treatment** | **Brown-Forsythe P-value** | |
| --- | --- | --- | --- |
|  |  | 3 Gy | 8 Gy |
| Hex-V-I | Abx-con | <0.001, 1d 3d | <0.001, 1d 3d |
|  | Abx | 0.009, 1d | 0.001, 3d |
| Creatine | Abx-con | 0.001, 3d | 0.014, 1d |
|  | Abx | 0.011, 1d | <0.001, 1d |
| Carnitine | Abx-con | 0.020, 1d | 0.001, 1d 3d |
|  | Abx | <0.001, 3d | 0.001, 1d 3d |
| TML | Abx-con | <0.001, 1d 3d | <0.001, 1d 3d |
|  | Abx | 0.004, 1d | 0.001, 1d |
| N1-Acetylspermidine | Abx-con | 0.017 | 0.005, 1d |
|  | Abx | 0.003, 1d | 0.050 |
| Citric acid | Abx-con | 0.007, 1d | <0.001, 1d 3d |
|  | Abx | 0.377 | 0.102 |
| Betaine | Abx-con | 0.179 | 0.002, 1d |
|  | Abx | 0.048 | 0.027 |
| Proline Betaine | Abx-con | 0.486 | 0.011, 3d |
|  | Abx | 0.842 | 0.031 |
| Acetyl-arginine | Abx-con | 0.002, 1d | <0.001, 1d 3d |
|  | Abx | 0.096 | 0.047 |
| *cis*-Aconitic acid | Abx-con | 0.019, 1d | 0.033, 1d |
|  | Abx | 0.123 | 0.054 |
| 307.2025_5.69 | Abx-con | <0.001, 1d 3d | <0.001, 3d |
|  | Abx | ND | ND |
| 347.1227_0.27 | Abx-con | <0.001, 1d | 0.001, 1d |
|  | Abx | 0.196 | 0.137 |

ND – Not Detected
