# Supplementary material for: Host microbiome depletion attenuates biofluid metabolite responses following radiation exposure
Source: PLoS One. 2024 May 17;19(5):e0300883. doi: 10.1371/journal.pone.0300883 (PMC11101107; doi:10.1371/journal.pone.0300883)
Supplement: S3 Table — (DOCX) [file pone.0300883.s003.docx]

**Supplementary Table 3.** Mixed effects model output including the regression parameters, standard errors, p-values, and diagnostics on regression residuals.

| Metabolite | Regression parameter estimates*, Std Error, degrees of freedom, t values, p values | Diagnostics on regression residuals: Skewness and Kurtosis ** | |
| --- | --- | --- | --- |
| Hex-V-I | 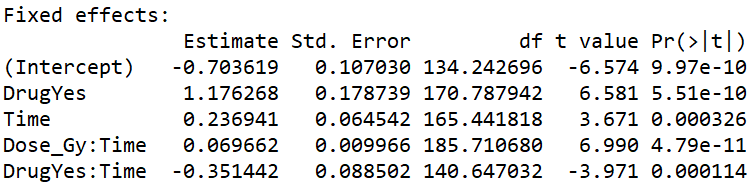 | -0.54 | 3.19 |
| Creatine | 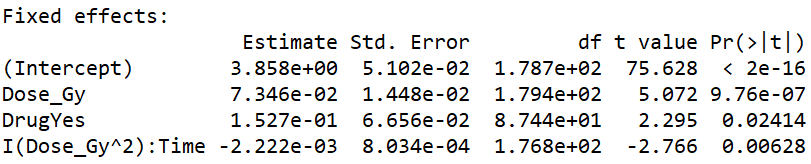 | 0.20 | 3.75 |
| Carnitine | 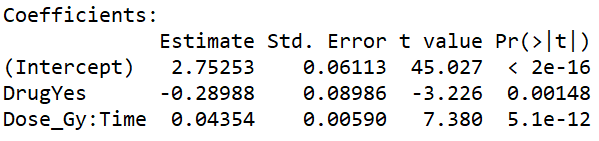 | 0.27 | 2.89 |
| TML | 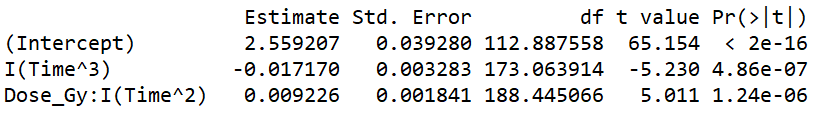 | -0.15 | 2.44 |
| N1-Acetylspermidine | 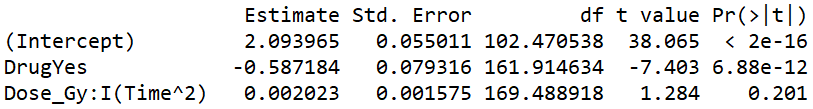 | 0.22 | 3.36 |
| Citric acid | 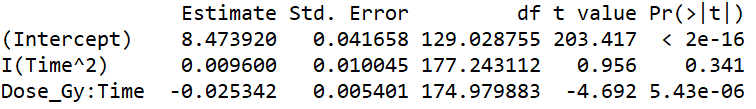 | 0.62 | 3.60 |

* These are the fixed effect parameters from the best-supported mixed effects model variant, selected by AICc among several variants.

** For the Normal distribution, Skewness should be 0 and Kurtosis should be 3. Values close to these suggest that the distribution of residuals is close to Normal.
