# Supplementary material for: Host microbiome depletion attenuates biofluid metabolite responses following radiation exposure
Source: PLoS One. 2024 May 17;19(5):e0300883. doi: 10.1371/journal.pone.0300883 (PMC11101107; doi:10.1371/journal.pone.0300883)
Supplement: S4 Table — (DOCX) [file pone.0300883.s004.docx]

**Supplementary Table 4.** P-values for serum metabolites that were significantly perturbed due to ionizing radiation exposure.

| **Metabolite** | **Treatment** | **Brown-Forsythe**  **P-value** |
| --- | --- | --- |
| Proline Betaine | Abx-con | 0.018 |
|  | Abx | 0.001 |
| Carnitine | Abx-con | 0.207 |
|  | Abx | 0.051 |
| Citric acid | Abx-con | <0.001 |
|  | Abx | <0.001 |
| LysoPC (14:0) | Abx-con | 0.001 |
|  | Abx | 0.013 |
| LysoPC (16:1) | Abx-con | 0.032 |
|  | Abx | 0.001 |
| LysoPC (18:3) | Abx-con | 0.038 |
|  | Abx | 0.208 |
| LysoPC (20:5) | Abx-con | 0.004 |
|  | Abx | 0.001 |
| LysoPC (20:3) | Abx-con | 0.005 |
|  | Abx | <0.001 |
| LysoPC (22:5) | Abx-con | 0.044 |
|  | Abx | 0.002 |
