# Supplementary figures and images for: Host microbiome depletion attenuates biofluid metabolite responses following radiation exposure
Source: PLoS One. 2024 May 17;19(5):e0300883. doi: 10.1371/journal.pone.0300883 (PMC11101107; doi:10.1371/journal.pone.0300883)

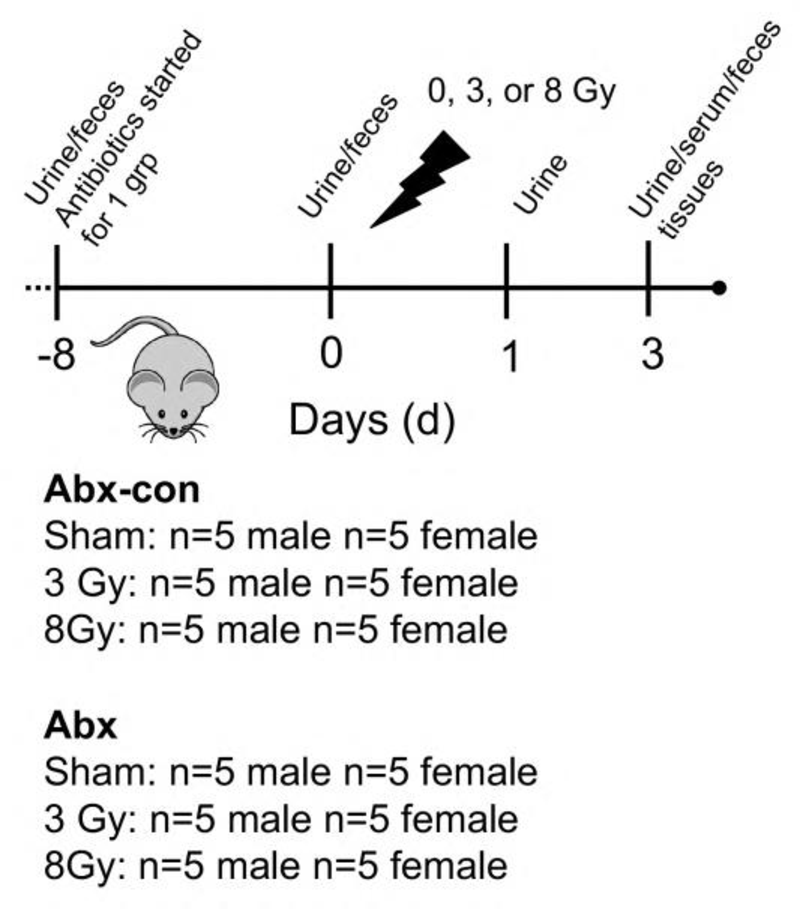

Supplement: S1 Fig — Urine and feces were collected from mice before they were given broad spectrum antibiotics for 8 days prior to exposure to ionizing radiation. Mice were exposed to either 0, 3, or 8 Gy ionizing radiation and urine was collected at days 0, 1, and 3 days and feces were collected at day 0 and 3. At the end of the experiment serum and tissues were collected post-mortem. (TIF) [file pone.0300883.s005.tif]

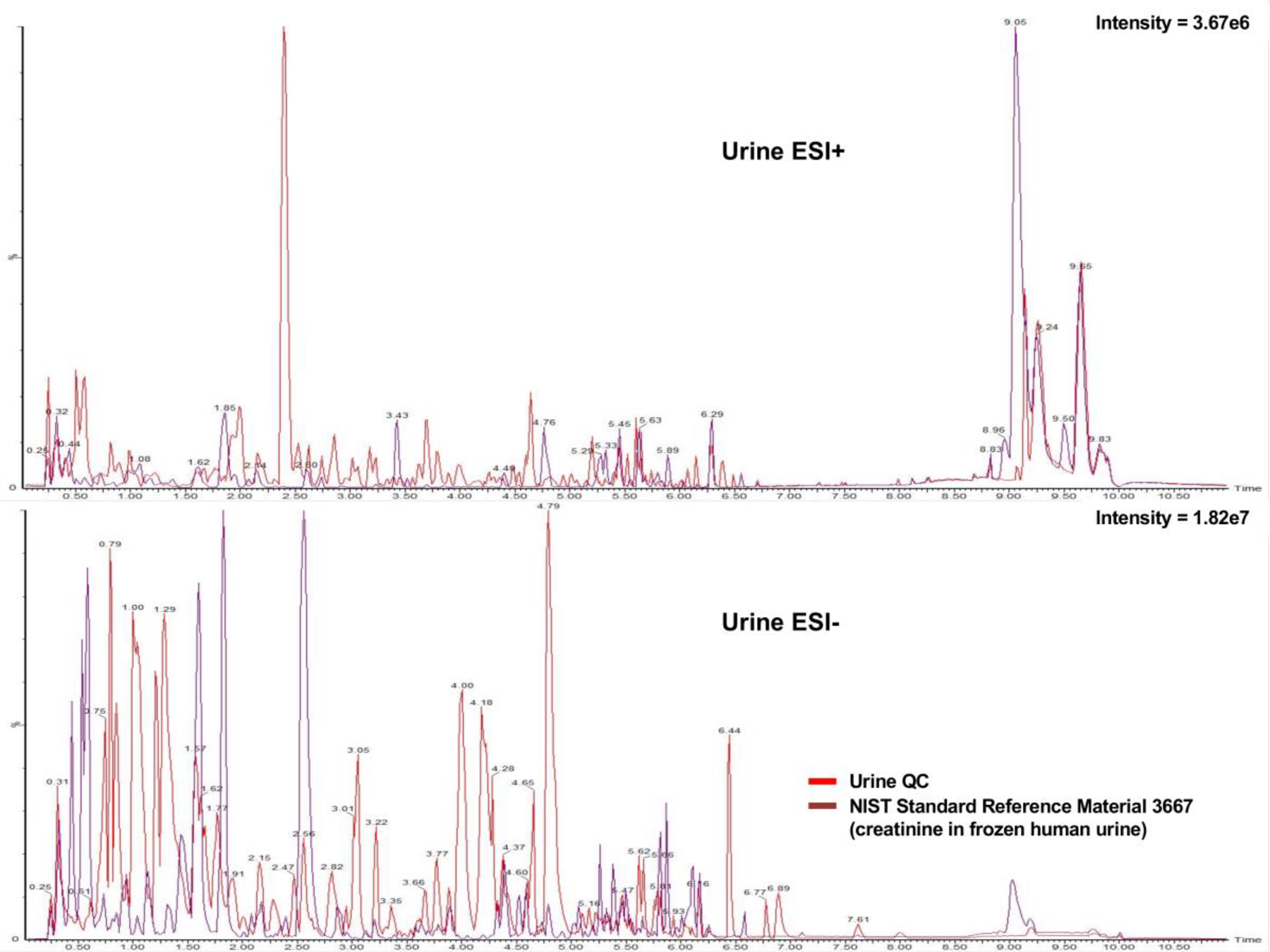

Supplement: S2 Fig — Base peak chromatograms in ESI+ (top) and ESI- (bottom) modes of the pooled urine control sample overlaid with the NIST Standard Reference Material 3667 (creatinine in frozen human urine). (TIF) [file pone.0300883.s006.tif]

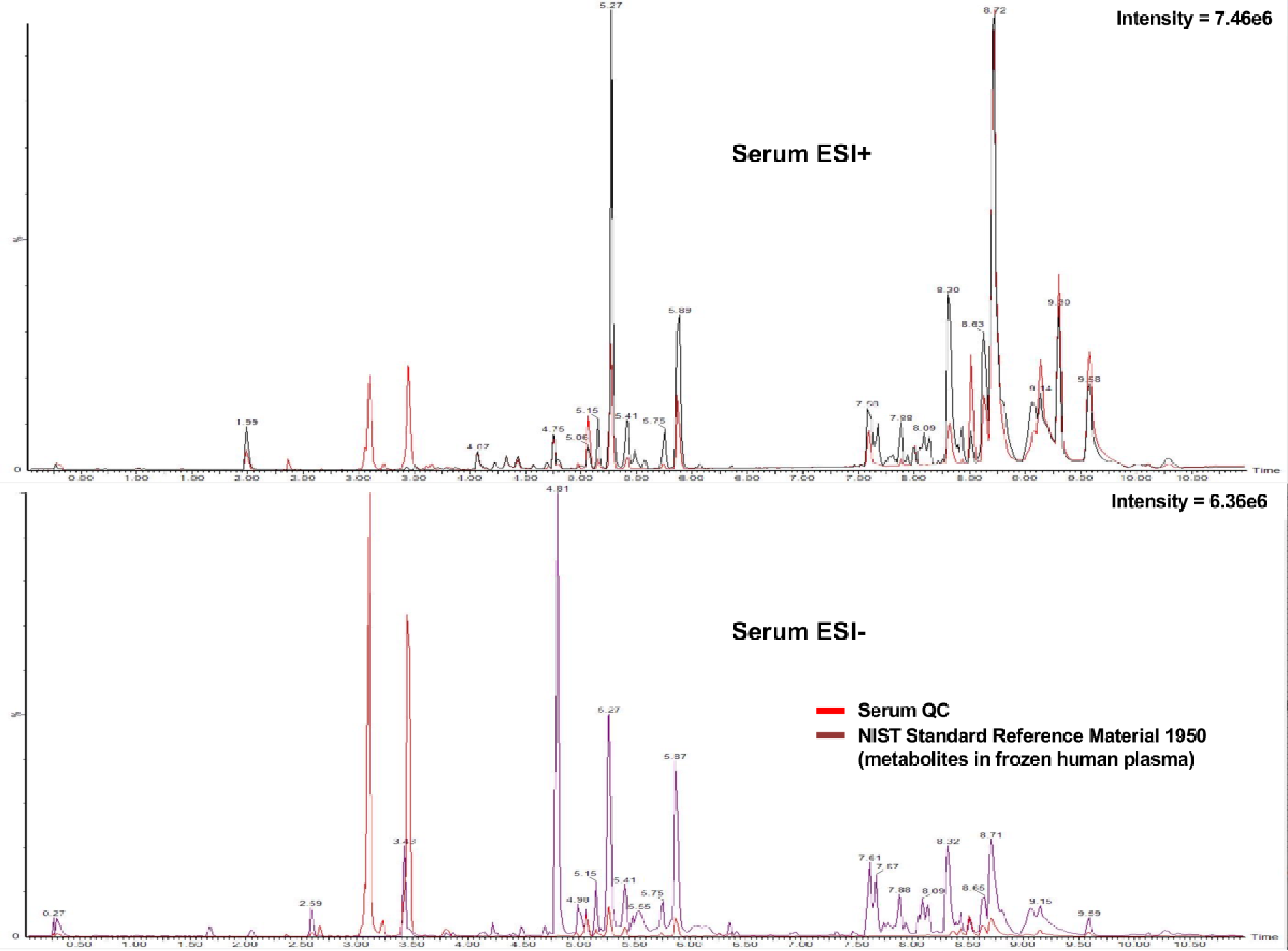

Supplement: S3 Fig — Base peak chromatograms in ESI+ (top) and ESI- (bottom) modes of the pooled serum control sample overlaid with the NIST Standard Reference Material 1950 (metabolites in frozen human plasma). (TIF) [file pone.0300883.s007.tif]

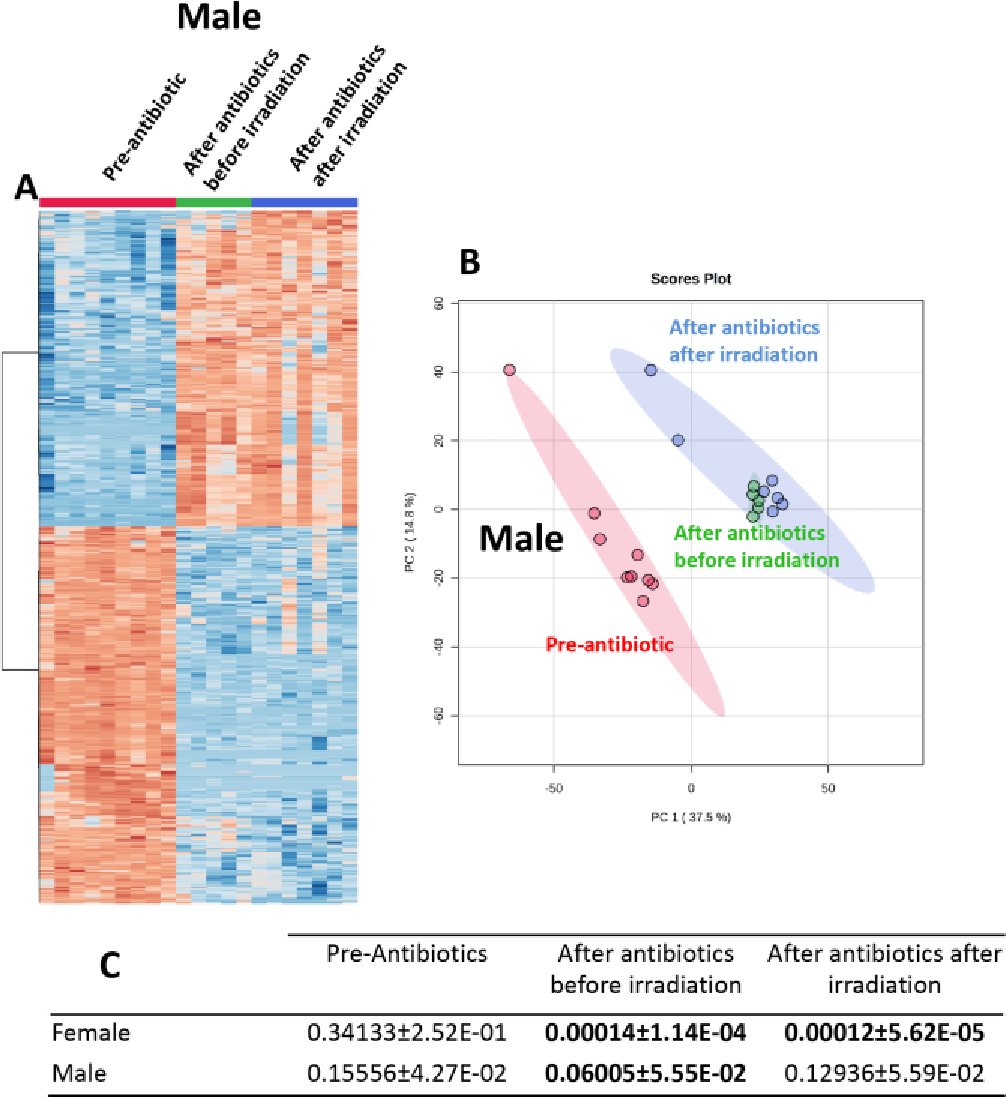

Supplement: S4 Fig — A) Heatmap produced of the top 250 urinary metabolites from male mice used for fecal DNA extraction in Table C. The heatmap shows similar profiles for mice after supplying broad-spectrum antibiotics in the drinking water in contrast to the DNA levels observed. B) Similarly, a PCA plot shows distinct differences in the urine metabolome between male mice before antibiotics vs. post-antibiotic administration. C) Table of the copy # of HV3-16S (x104) / 1 mg fecal mass for male and female mice before antibiotic administration, after antibiotic administration but before irradiation, and after antibiotic administration and irradiation. Bold text indicates statistical significance from a one-way ANOVA P < 0.05. (TIF) [file pone.0300883.s008.tif]

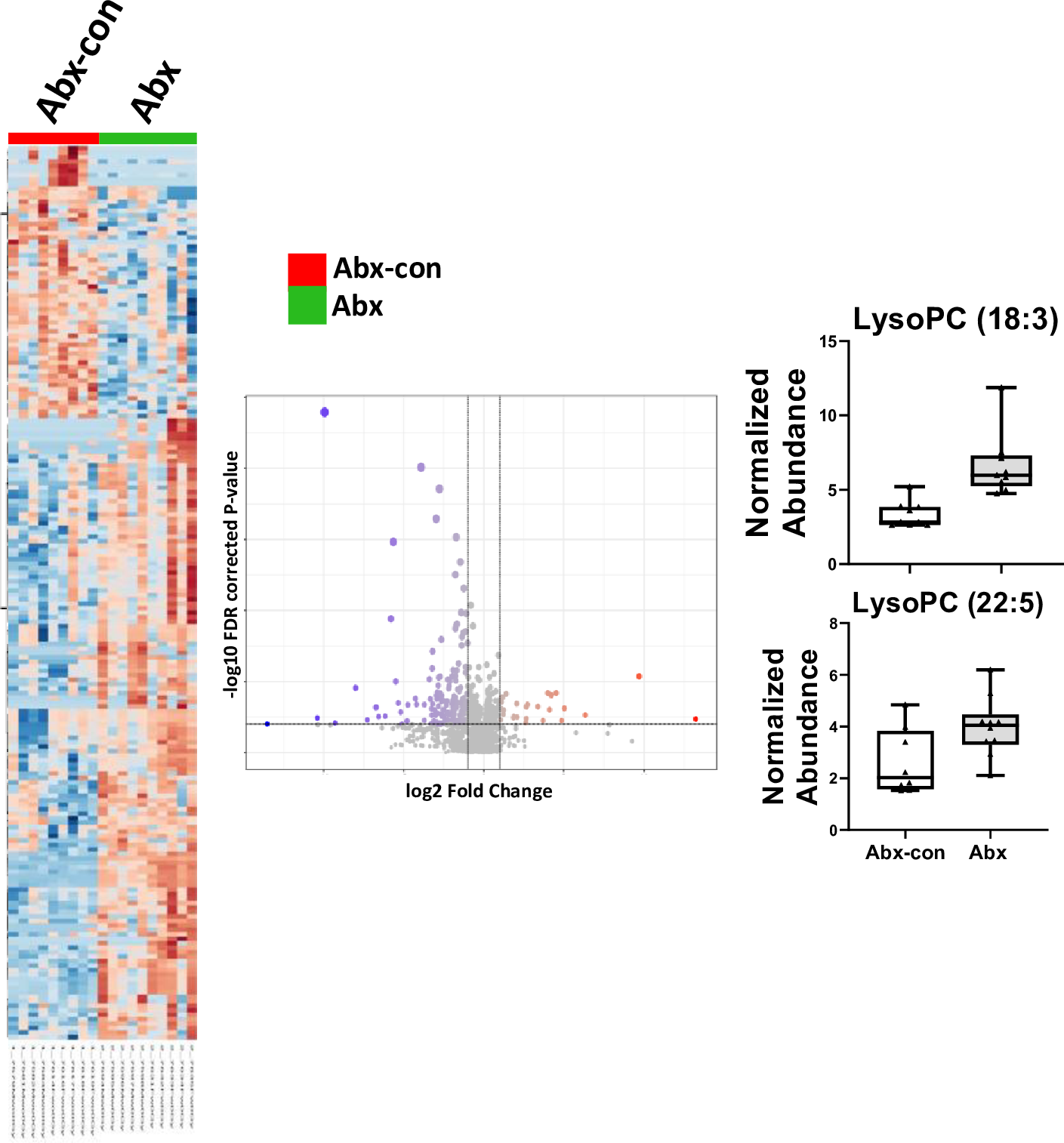

Supplement: S5 Fig — After removal of the host microflora, 10.2% of serum spectral features (primarily corresponding to lipids) showed significantly different levels as shown in above heatmap and volcano plot generated from the positive mode ions in MetaboAnalyst. (TIF) [file pone.0300883.s009.tif]

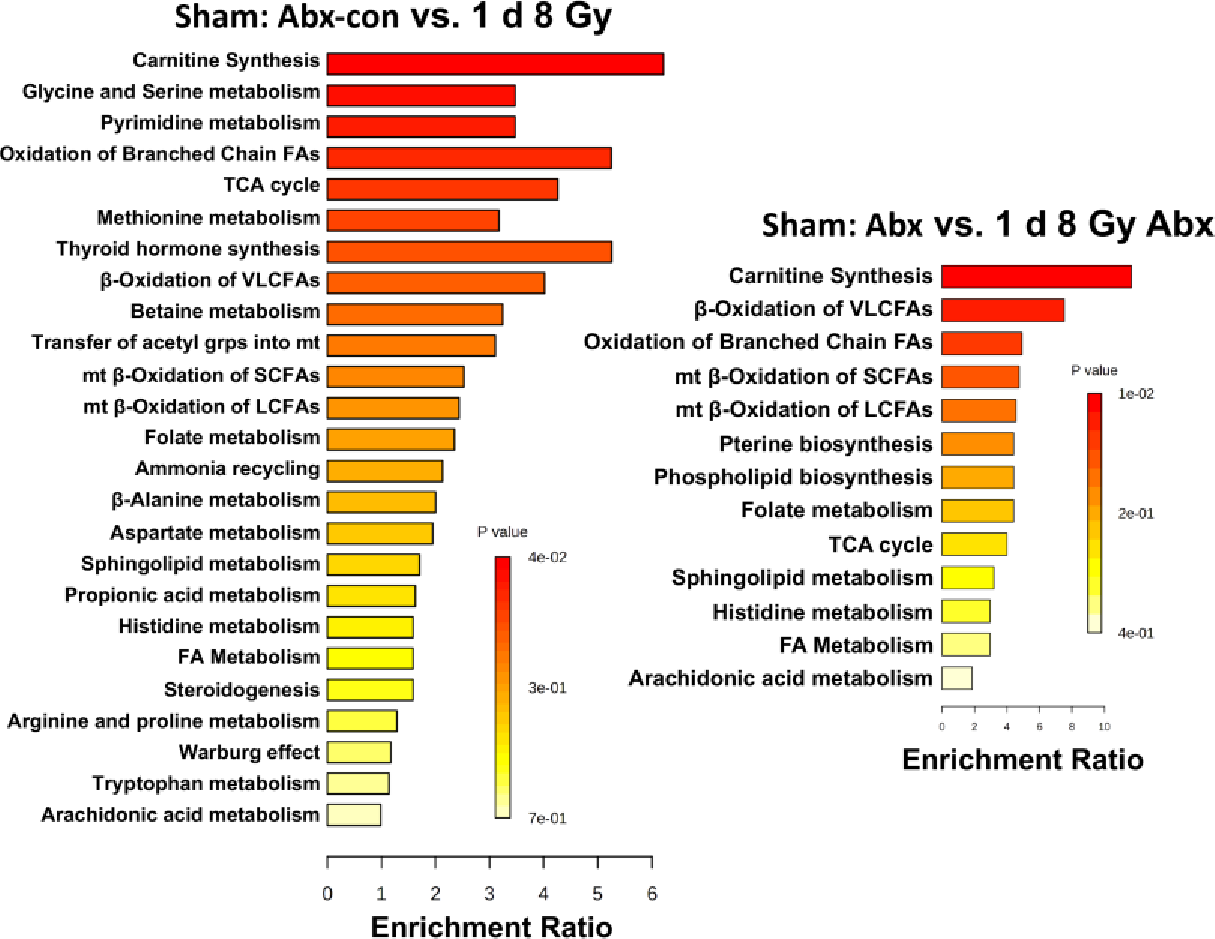

Supplement: S6 Fig — The significant metabolites generated from the volcano plot of the positive mode ions was used for enrichment analysis using the small molecule pathway database (SMPD). Mice with depleted host microflora had an absence of perturbation in pathways involved in some amino acid pathways (tryptophan, glycine, serine, methionine, aspartate, arginine, β-alanine, proline), pyrimidine metabolism, propionic acid metabolism, hormone synthesis, and ammonia recycling. Perturbation to the carnitine synthesis, TCA cycle, and mitochondrial beta-oxidation were conserved between treatments. (TIF) [file pone.0300883.s010.tif]

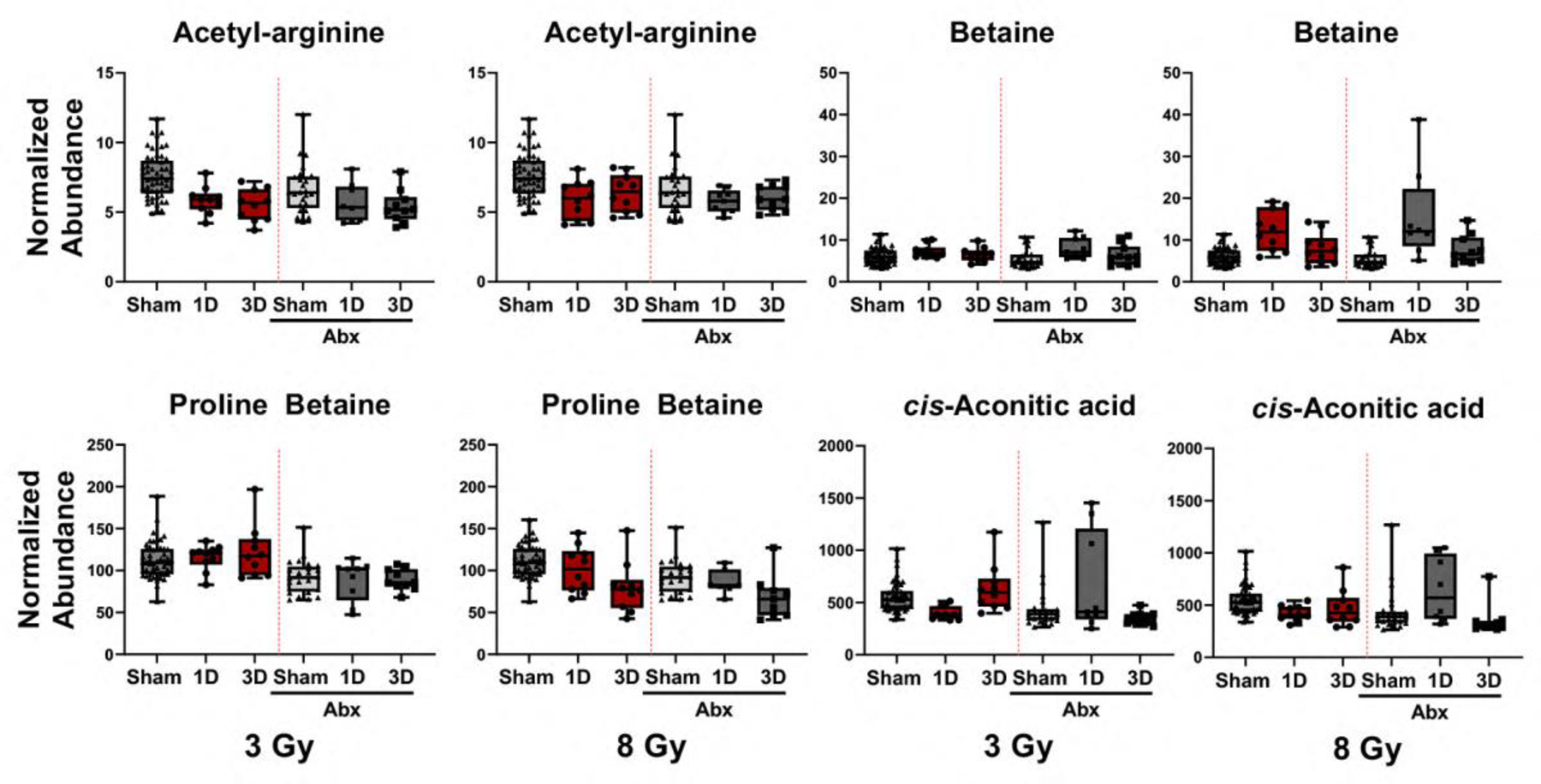

Supplement: S7 Fig — Metabolites with statistically significant changes from radiation exposure but were not used for constructing ROC curves in the current study. (TIF) [file pone.0300883.s011.tif]

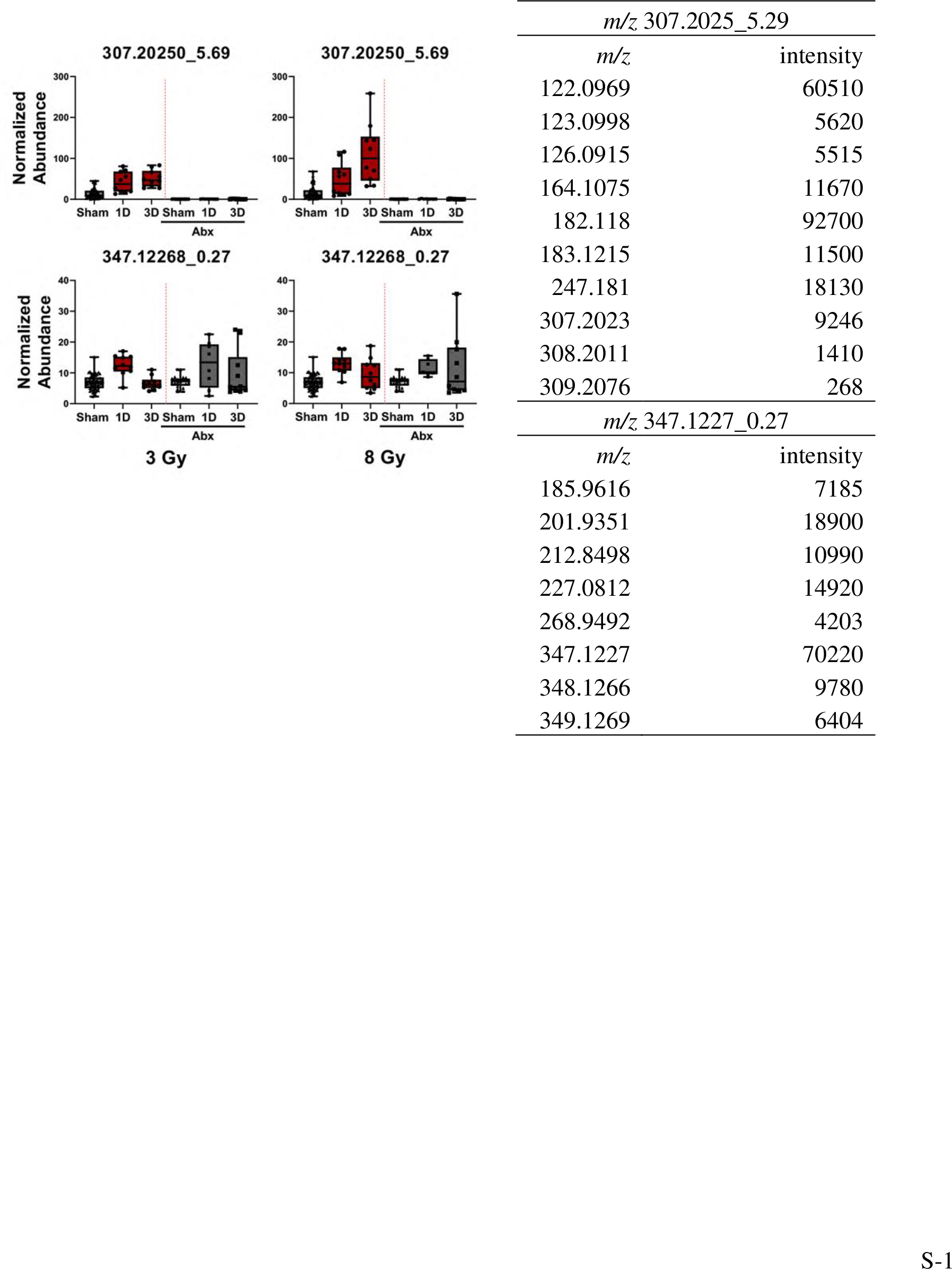

Supplement: S8 Fig — Tandem MS tables and concentrations for m/z 307.2025_5.29 and 347.1227_0.27 and concentrations post-irradiation. (TIF) [file pone.0300883.s012.tif]
