# Supplementary material for: Host microbiome depletion attenuates biofluid metabolite responses following radiation exposure
Source: PLoS One. 2024 May 17;19(5):e0300883. doi: 10.1371/journal.pone.0300883 (PMC11101107; doi:10.1371/journal.pone.0300883)
Supplement: Attachment — Submitted filename: Response to Reviewers.docx [file pone.0300883.s013.docx]

**We would like to thank the referees for taking the time to provide constructive criticisms and detailed review of this manuscript. The suggestions and critiques were addressed accordingly and ultimately strengthened the manuscript. Please find our responses listed below and bolded. Changes in the manuscript are marked with the track changes function in word and highlighted.**

**RESPONSE TO THE REVIEWERS:**

Reviewer #1: 1. In this manuscript, the authors reported their investigations on the effects of microbiome depletion on metabolomics biodosimetry. This research involves a lot of work and has novel methods. It is of great value for in dose reconstruction and novel biodosimetry assay. However, some revisions were required before publication.

**We would like to thank the reviewer for their kind comment.**

2. Page 12, metabolites with FDR corrected P value of <0.10 in urine or P value <0.20 in serum were selected. Except FDR, VIP is widely accepted and expected as a standard in metabolomics analysis, why VIP was not chosen for differentially metabolites definition? And what is the reason that using different P value in urine and serum?

**The FDR corrected P values are primarily used for filtering purposes on the initial post-processed data matrix. We observe lower fold changes in serum metabolites post-IR so we use a less stringent FDR corrected P value so we don’t filter out potentially important metabolites. After the initial data mining we still go through a validations stage and visually assess each metabolite. Here, we additionally used multimodel inference (MMI) techniques and linear regression models to check which urinary metabolites were the strongest predictors.**

**VIP is used as a filtering technique as well and we use it for Random Forests classification accuracy, but for the current study we were able to pick out all of the validated metabolites using the FDR P value screening using our in house MetaboLyzer software.**

3. Page 15, the results of sex specific differences in microbiome depletion efficiency should be noteworthy and further discussed.

**We agree that this could be a useful reference for others that want to repeat this design. A paragraph (results paragraph 1) and 3 figures (Fig 1, 2, and S4 Fig) have been used to discuss this unexpected result. We further added in a limitations paragraph at the end of the discussion (page 31, line 15) that states further studies may determine which microbes continued to proliferate in males after such a high antibiotic regimen, as the metabolome was clearly affected, but that a microbiome analysis was beyond the scope of the current study.**

**We also added citations to the discussion (Page 28 line 1) that discusses sex differences in both the stool and gut microbiome of Sprague Dawley rats given broad spectrum antibiotics and general sex differences in the basal microbiome.**

**Koliada, A., Moseiko, V., Romanenko, M. *et al.* Sex differences in the phylum‐level human gut microbiota composition. *BMC Microbiol* 21, 131 (2021).**

**Parodi, Gonzalo, et al. "The Response of the Rodent Gut Microbiome to Broad-Spectrum Antibiotics Is Different in Males and Females." *Frontiers in Microbiology* 13 (2022): 897283.**

4. Fig 4. The ROC curves of metabolite panel (Six or five biomarkers) were used to classify the cluster between irradiation and control. Some biomarkers, such as citric acid and N1-acetylspermidine, have lager error bar in several groups, especially in controls. The information on individual differences of the involved metabolite should be considered and discussed.

**In part of the reason the error bars are so large is that we are using the min-max setting instead of SD or SEM, but we feel that it is important to show each individual data point.**

**But more importantly, it is this natural individual variation that is driving the motivation of this study and several others from our group. There will be natural variation due to genetic, dietary, sex, etc.. differences, which will be compounded when going from C57Bl/6 mice to a human population. Also, a pre-irradiation urine sample will not be available to test each individual in a radiological emergency. These challenges were discussed in the introduction on page 3 line 20. We also added that this natural variation is likely why there is not a “silver bullet” in very few biological assays and that it will require integration of biomarkers from different specialties (page 19 line 3). The ultimate goal is to find genes, proteins, metabolites, or other markers that are radiation specific to build a biodosimetry assay.**

5. Fig 5. and Fig 7. There were not enough evidences that using the ROC panel to evaluate the real accuracy rate for other blind samples, although the AUROC > 0.90, that should be noteworthy and discussed.

**While we did not have training and validation cohorts in our current study, the metabolite panels have been identified in previous studies of ours. We had already included or added additional clarification at page 4 line 15, page 18 line 25, page 23 line 23, and also highlighted the novel aspects of this study at Page 6 line 17.**

Reviewer #2: The authors present a well-written manuscript on the effects of microbiome depletion with regards to radiation biodosimetry metabolomics. The data are interesting but as a non-expert in the field I found it challenging to identify the research question being asked. There were a number of other areas where the manuscript could be improved to aid clarity. I also have some questions about the statistical approach and whether it is appropriate to the question being asked.

RESEARCH QUESTION
As a non-expert. this was not clear to me. I believe that it may be "Is depletion of the microbiome a confounder for radiation biodosimetry by metabolomics". If this is the case, I suggest a more positive title, rather than the current descriptive one (it is usually not a good idea to have a title that says that there is an effect, e.g. from microbiome depletion, without letting the reader know what those effects are, especially when the article concludes that there is a panel resistant to this confounder).

**As stated below, we have added a purpose statement of the current study to the introduction (Page 5 Line 19) and clarified the novelty of the current study (Page 6 line 17).**

**We have changed the title to “Host Microbiome Depletion Attenuates Biofluid Metabolite Responses Following Radiation Exposure” to more accurately reflect the subject material.**

FIGURES

The figures are generally poorly put together and would benefit from substantial improvement.

For example, Figures 1D and 1C are too small and the labelling of the points is illegible. I cannot be sure, but it looks as if the m/z values and retention times are presented - are there no metabolite names? Whilst I appreciate that it is hard to match font sizes in multi-part figures exactly, the PCA and volcano plot have completely different axis labels, and the former are so small as to be unreadable. It does not help the reader.

**The PCA and volcano plot were generated by MetaboAnalyst as a way to simply show separation of groups and the spread of statistically significant spectral features. The labelling on the axes has been fixed. The individual labels on the volcano plot were removed.**

As another example, Figure 2, why drop the 2A and 2B convention from the rest of the paper? It is better to be consistent. Also, the Venn diagram in Figure 2 (Left, Bottom) is poor, the overlap of 241 is BY FAR the largest part and is shown as the smallest part of the Venn diagram (and also doesn't follow the colour scheme of Figure 2 (Left, Top)).

**The Venn diagram was automatically generated by an online program, metorigin, and we agree that it does not add any additional information to the figure as it is the same in Figure 2 (Left, Top). Figure 2 (Left, Bottom) has been removed. The figure has been split into an A and B panel and labeled to help orient the reader.**

Another example, Figure 3, the volcano plots are once again so small as to be pointless - what can the reader deduce from the illustrations in an efficient manner? I couldn't gain any additional information.

**As stated above, the volcano plots are simply to illustrate the number of significantly different spectral features and not to identify each feature. We have removed the labels on the individual data points.**

Also, multi-part figures should each have their own lettering, it is not best practice to just say Figure 3A and include three separate charts in Figure 3A.

**The A and B subheadings are to differentiate panels between the control group and the mice with a depleted microbiome. Within each panel is a heatmap, volcano plot, and a PCA plot, which are rather standard figures to include for -omics datasets. We understand that in the text it is not entirely clear which subparts of the figure we are referring to so we have clarified if the heatmap, volcano plot, or PCA plot is being discussed (Pg 17 line 14-17).**

It is very helpful to the reader of an article, especially a non-expert, to have clearly labelled and captioned Figures

**We apologize that the figures were confusing, we have made all suggested edits to the figures and they should be easier to follow.**

Please also be consistent, don't use Figure 3, and then Fig 6.

**This typo has been corrected.**

INTRODUCTION AND STATISTICAL APPROACH

As a non-expert I found the paper challenging to read, but I believe that the topic deals with determining radiation exposure by means of biodosimetry assays. I found that the Introduction was poor in explaining the background to this. Metabolomics is often not the first-line solution to problems, as it involves additional instrumentation. I did not understand why metabolomics was selected in preference to other tools, and would like the Introduction to better explain what UNMET NEED in the field of biodosimetry is being met here. I would have appreciated more discussion of other methods and why they are inadequate to the task, in order to understand the importance of THIS research. For example:

New Approaches for Quantitative Reconstruction of Radiation Dose in Human Blood Cells
DOI: 10.1038/s41598-019-54967-5

Biomarkers to Predict Lethal Radiation Injury to the Rat Lung
doi: 10.3390/ijms24065627

These may not be the correct articles to cite, but the Introduction must be improved to demonstrate the context and why the research is useful, there is only brief mention of previous research.

**We thank the reviewer for pointing out that the introduction was not descriptive enough. The reason metabolomics is chosen is because our group is a designated Centers for Medical Countermeasures against Radiation that is funded through NIAID. Within this there are three projects that specialize using genomics, transcriptomics, and metabolomics with the common goal of improved high-throughput biodosimetry. The paper cited above (DOI: 10.1038/s41598-019-54967-5) was from our transcriptomic group, while we are the metabolomics section of the Center. Our primary person in the statistics core (Igor Shuryak), who is co-author on the above paper and this study, attempts to integrate the results from the three projects into a multi-omics approach for biodosimetry. So, it is not that any method is inadequate, it’s that each method has its strengths (such as metabolomics is the only method for measuring radiation responses in urine) and the idea is to combine their strengths into a single assay.**

**We have added a purpose statement of the current study to the introduction (Page 5 Line 19), clarified the novelty of the current study (Page 6 line 17), and added a recent review that thoroughly outlines the goals and mission of the Radiation and Nuclear Countermeasures Program within NIAID (Page 4 line 16). This review is open access and was recently published in Sept of 2023.**

**Satyamitra, M. M., Cassatt, D. R., Molinar-Inglis, O., Rios, C. I., Taliaferro, L. P., Winters, T. A., & DiCarlo, A. L. (2023). The NIAID/RNCP Biodosimetry Program: An Overview. *Cytogenetic and Genome Research*.**

Also, AUROC and PCA are not that helpful in regression problems, these are typically more useful in classification problems. If the goal is to develop a biodosimetry assay, then the appropriate statistical reporting should include assessment of regression accuracy on the sham and Abx mice, and demonstrate e.g. the RMSE, goodness of fit of the model, examine linearity. If these things cannot be done, this is a severe limitation of the study, and must be reflected in the text, because at the moment I was not at all clear that you have demonstrated that the models you have built can be used to accurately measure the dose received by the mice, and what the errors are (including error bars on the estimates, of course).

**The point of including the PCA plots is to show that there was a higher separation between the control and irradiated groups for Abx-con mice vs. Abx mice. This ties in with the rest of the paper that there is a high influence of the host microbiome on biofluid metabolomic profiles and that by removing it you will reduce the magnitude of change after any stress response, not just from radiation responses. This is simply a common visualization technique in addition to the heatmaps and volcano plots.**

**In addition, the mixed effects model output including the regression parameters, standard errors, p-values, and diagnostics on regression residuals have been supplied in Supplementary Table 3, (page 21, line 17).**

Overall, the Introduction and Discussion both need to take account of previous work, the advantages of the proposed method, and a proper statistical demonstration of the accuracy of the proposed method and reconstructing the dose with a proper assessment of the goodness of fit. Alternatively, it would be necessary to explain why PCA is preferred to other methods of assessing dose reconstruction.

**The purpose of this study was to determine how removing microbial metabolites may affect metabolomic profiles that have been established by us in the lab. Therefore, we chose relevant doses (3 and 8 Gy) and time points (1 and 3 d) that have been used before and added the explanation below on page 7 line 9:**

**“The doses and times chosen represent priority groups after a nuclear emergency, as the dose equivalent in humans will be zero damage (sham), minor damage (3 Gy), and major damage (8 Gy, an ~LD_50/30_ value for C57Bl/6 mice). An optimistic expectation would to have all individuals screened within 3 d, however, following the panic ensuing a nuclear emergency we realize that this may take longer.”**

**We added the more immediate need of the current study is to determine if the contribution and variation within the host microbiome will negatively impact our established metabolite panel and that that a final assay capable of comprehensive of dose reconstruction is beyond the scope of this study because only 2 doses and a sham were performed (page 31 line 23).**

Figure 6A, what is the statistical basis for the ellipses added, why are they not added to Figure 6B? Why are the ellipses in 2 dimensions on a 3D chart? Why does the green ellipse not include the outlier, whereas the red ellipse does include the outlier? If ellipses are to be added, this is best done by Hotellings or similar.

**The point of Fig 6 was to show there was a higher separation between the control and 8 Gy groups at the 3d time point for Abx-con mice vs. Abx mice. We removed the shapes drawn in ppt and replaced them with the MetaboAnalyst 95% CI overlays.**

MINOR COMMENTS

Abstract Line 8 Minor comment, as a non-expert I would find "Here, we 'knocked out' the microbiome of ... mice using antibiotics (Abx mice)' more helpful.

**We have made the change in wording.**

Introduction Line 7, why have you added an acronym for IND, when IND is never again referred to in the text?

**We removed the acronym IND.**

Line 15, remove the word 'incorporation' or state what these assays are being incorporated to

**We have made the change in wording.**

There is no Limitations paragraph in Discussion - this omission should be addressed.

**A paragraph discussing limitations of the study was added (Page 31 line 15).**
